# Investigations on Na+, K+-ATPase energy consumption in ion flow of hydrophilic pores by THz unipolar stimulation

**DOI:** 10.1016/j.isci.2023.107849

**Published:** 2023-09-07

**Authors:** Wenfei Bo, Rong Che, Qiang Liu, Xiaobo Zhang, Yintao Hou, Yubin Gong

**Affiliations:** 1 College of Information and Communication, National University of Defense Technology, Wuhan 430000, China; 2 School of Electronic Science and Engineering, University of Electronic Science and Technology of China, Chengdu 610054, China

**Keywords:** Cellular physiology, Cellular neuroscience, Cell biology

## Abstract

Terahertz science and technology has recently shown new application prospects in artificial intelligence. It is found that terahertz unipolar stimulation can activate cell membrane hydrophilic pores. However, the behaviors of Na+, K+-ATPase and energy consumption during this period remain unknown. This paper investigates these behaviors by Na+, K+-ATPase and electroporation models, based on the interaction theory between terahertz fields and ions at the cellular level. The effective diameters of life ions are considered in the aqueous solution. From results, Na+, K+-ATPases can be activated and stay for a while before close after the stimulation. Their life ion flows are far lower than the flows via the pores. And their power dissipation is as low as 10^−11^ W in both rat neostriatal neurons and guinea pig ventricular myocytes. The results keep tenable in 0.1–1.2 THz. These lay the basis for investigations of information communication mechanisms in cells under terahertz stimulation.

## Highlights

• Na+, K+-ATPases can be activated under terahertz unipolar stimuli
• The ATPase and hydrophilic pores can stay open before close after the stimuli
• Flow via the ATPase is much smaller than that via the pores under the stimuli
• Power dissipation of the ATPase during flow via pores is extremely low in a cell

## Graphical abstract

**Figure:**
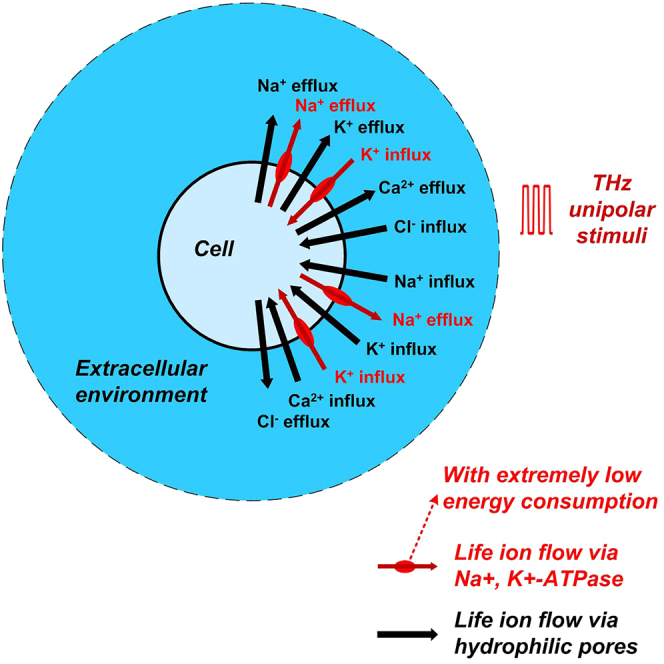

## Introduction

Currently, the development in terahertz science and technology has shown that terahertz electromagnetic waves can be generated, transmitted, and activate cell membrane voltage-gated ion channels in nerve cells.^1^^,^^2^^,^^3^^,^^4^^,^^5^ Especially, terahertz waves can transmit in the myelin sheath on the nerve axon membrane, and the electromagnetic energy can be supplemented and amplified at the nodes of Ranvier between two adjacent myelin sheaths.^1^ As the frequency band of terahertz electromagnetic waves is several orders of magnitude larger than that of current mobile communication, it implicates the approaching to the reveal of the mechanism of high-speed information transmission and processing of large-scale intelligent data by human brain, which is unable to be achieved at 4-G or 5-G communication frequency.^6^^,^^7^^,^^8^ Those show new important scientific and application values in one of the frontier development directions in the research of artificial intelligence (AI), i.e., terahertz-rate noninvasive brain-machine communication, and also the values in the frontier development of AI inspired by biological brain.

Terahertz stimulation can cause the transmembrane flow of life ions and thus lead to the information communication of cells.^3^^,^^4^^,^^9^^,^^10^^,^^11^^,^^12^^,^^13^^,^^14^^,^^15^ When the information communication takes place, the transmembrane flow of life ions leads to the deviation of ion concentrations of those life ions from their physiological equilibrium concentrations.

Na+, K+-ATPase is a type of membrane protein that consumes the cell metabolic energy to make the life Na+ and K+ ion concentrations back to their physiological equilibrium concentrations and preserves and supplies the energy for the information communication to which Na+ and K+ ions are closely correlated.^16^ Recently, it is found that terahertz unipolar picosecond (ps) pulse train stimulation can activate cell membrane hydrophilic pores,^17^^,^^18^ which also leads to the transmembrane flow of life ions, like the flow of Na+ and K+.^18^^,^^19^ The transmembrane flow of life ions via the cell membrane hydrophilic pores would influence the whole-cell transmembrane flow of life ions and thus possibly has impact on the information communication of cells.^18^^,^^19^^,^^20^ However, it is unknown whether Na+, K+-ATPase would be activated and how much the power dissipation of Na+, K+-ATPase is from cell metabolic energy during the transmembrane flow of Na+ and K+ ions via cell membrane hydrophilic pores by the terahertz unipolar stimulation.

This paper theoretically studies the behaviors and the energy consumption of Na+, K+-ATPase during life ion flow via cell membrane hydrophilic pores under terahertz unipolar picosecond pulse train stimulation. The influence of the hydrophilic pores on the information communication under the stimulation is studied from the perspective of energy dissipation.

## Results

The cell is under the stimulation of terahertz unipolar picosecond pulse train for a few nanoseconds and then the stimulation is turned off. After the stimulation, the numerical calculations of transmembrane life ion flow and energy dissipation continue for around 10 times longer, so as to study the behaviors and power dissipations of Na+, K+-ATPase.

**Figure 1:**
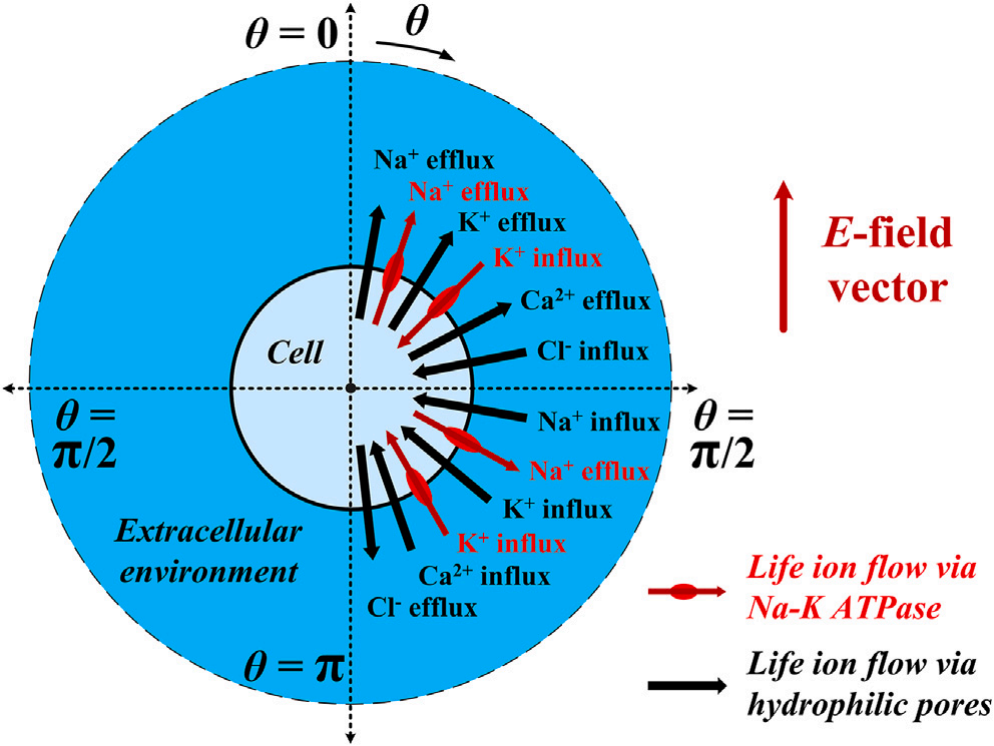
Illustration of cell system under the stimulation of terahertz unipolar picosecond pulse train The cell and extracellular environment are marked. And the electric field (E-field) vector of the pulse train is depicted in the image. The polar angle **θ** in the spherical coordinate system is marked and defined. The Na^+^, K^+^, Ca^2+^, and Cl^−^ flows via cell membrane Na+, K+-ATPase and hydrophilic pores as well as their directions are respectively schematically illustrated in the image.

**Figure 2:**
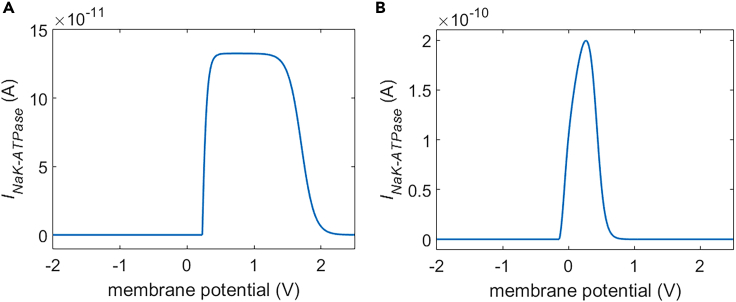
The current-voltage (I-V) response characteristics of cell membrane Na+, K+-ATPase in different cell types at the initial ion concentrations (*c*_*Na_i*_ = 12 mol/m^3^, *c*_*Na_o*_ = 145 mol/m^3^, *c*_*K_i*_ = 139 mol/m^3^, *c*_*K_o*_ = 4 mol/m^3^) (A and B) (A) I-V characteristics in rat neostriatal neuron; (B) I-V characteristics in guinea pig ventricular myocyte.

The illustration of cell system under the stimulation is shown in Figure 1. And the current-voltage (I-V) response characteristics of Na+, K+-ATPase in rat neostriatal neuron and guinea pig ventricular myocyte are respectively shown in Figures 2A and 2B at the initial ion concentrations. The details are depicted in STAR methods.

### Cell life ion flow and Na+, K+-ATPase power dissipation under the stimulation

#### Life ion flows via Na+, K+-ATPase and hydrophilic pores

**Figure 3:**
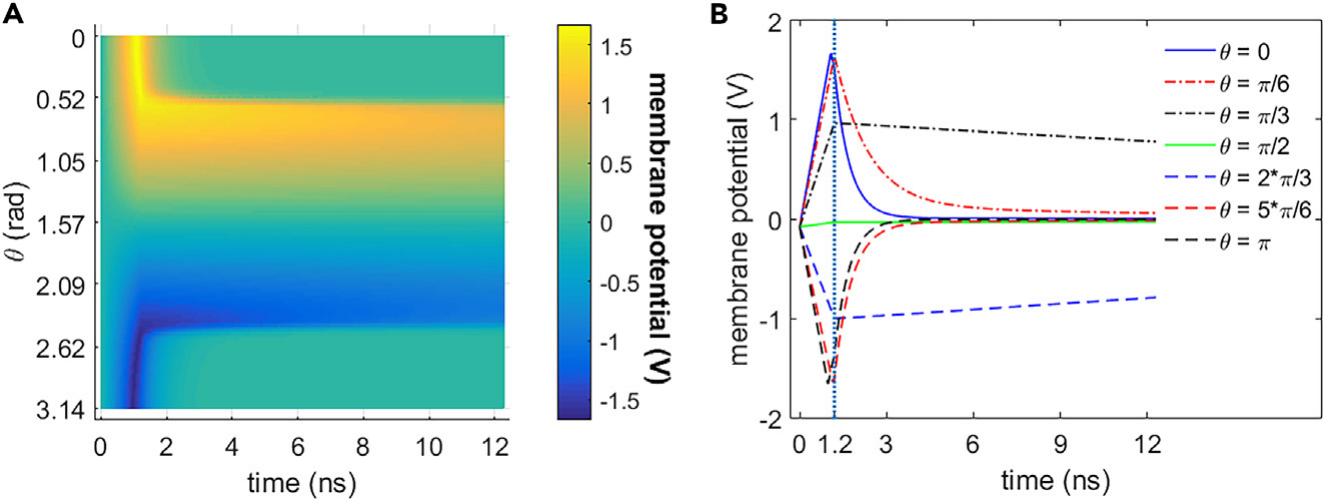
Membrane potential of rat neostriatal neuron versus time under the 1.2 ns stimulation of 5 × 10^7^ V/m, 0.5 THz, 1 ps unipolar pulse train After the stimulation, the numerical calculations continue for around 11 ns. (A) Membrane potential with respect to polar angle ***θ*** versus time. (B) Membrane potential at ***θ*** = 0, ***π***/6, ***π***/3, ***π***/2, 2***π***/3, 5***π***/6, and ***π*** versus time.

**Figure 4:**
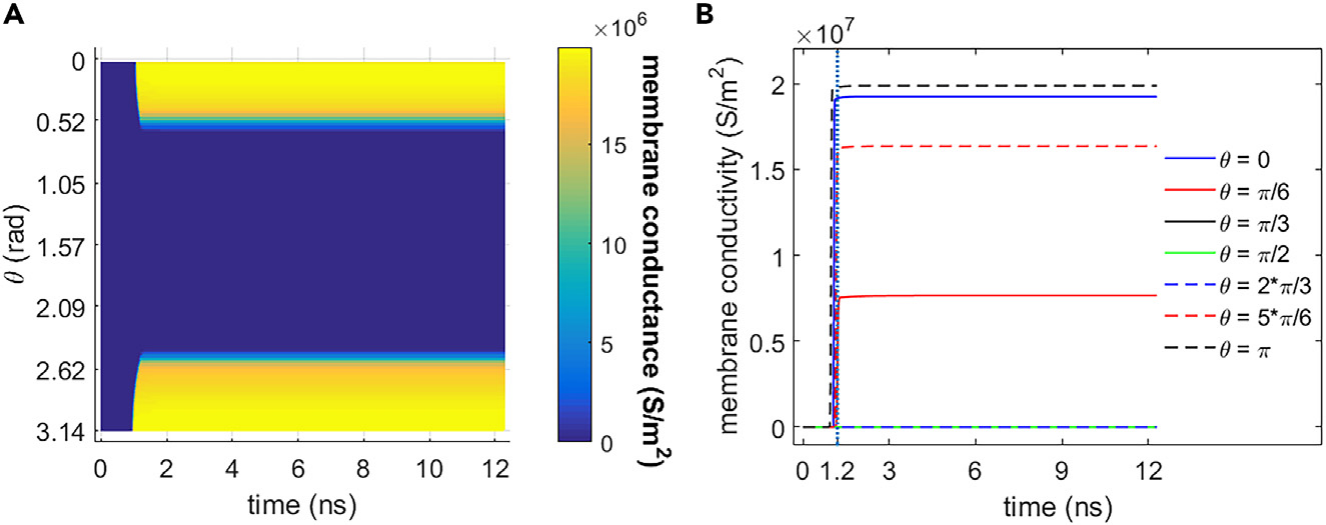
Membrane conductivity of rat neostriatal neuron versus time under the 1.2 ns stimulation of 5 × 10^7^ V/m, 0.5 THz, 1 ps unipolar pulse train. After the stimulation, the numerical calculations continue for around 11 ns (A) Membrane conductivity with respect to polar angle ***θ*** versus time. (B) Membrane conductivity at ***θ*** = 0, ***π***/6, ***π***/3, ***π***/2, 2***π***/3, 5***π***/6, and ***π*** versus time.

As shown in Figures 3 and 4, 0.5 THz, 1 ps unipolar pulse train stimulation activates cell membrane hydrophilic pores in rat neostriatal neuron during the 1.2 ns stimulation. And the hydrophilic pores stay at least 11 ns after the shutdown of the stimulation. During the stimulation that is from 0 to 1.2 ns, the absolute values of the membrane potential at each polar angle θ except θ = π/2 increase significantly before the activation of hydrophilic pores at around 0.94 ns and then start to decrease. The reason for the exception at θ = π/2 is that cell membrane is parallel to the stimulated terahertz electric field at this polar angle. The membrane conductivities keep zero before the activation of hydrophilic pores and increase significantly after the activation and finally tend to be nearly stable. After the stimulation (>1.2 ns), the absolute values of membrane potentials decrease significantly, and the membrane conductivities keep almost stable in around 11 ns. It means that the hydrophilic pores activated in cell membrane during the stimulation stay stable in at least 11 ns after the stimulation.

**Figure 5:**
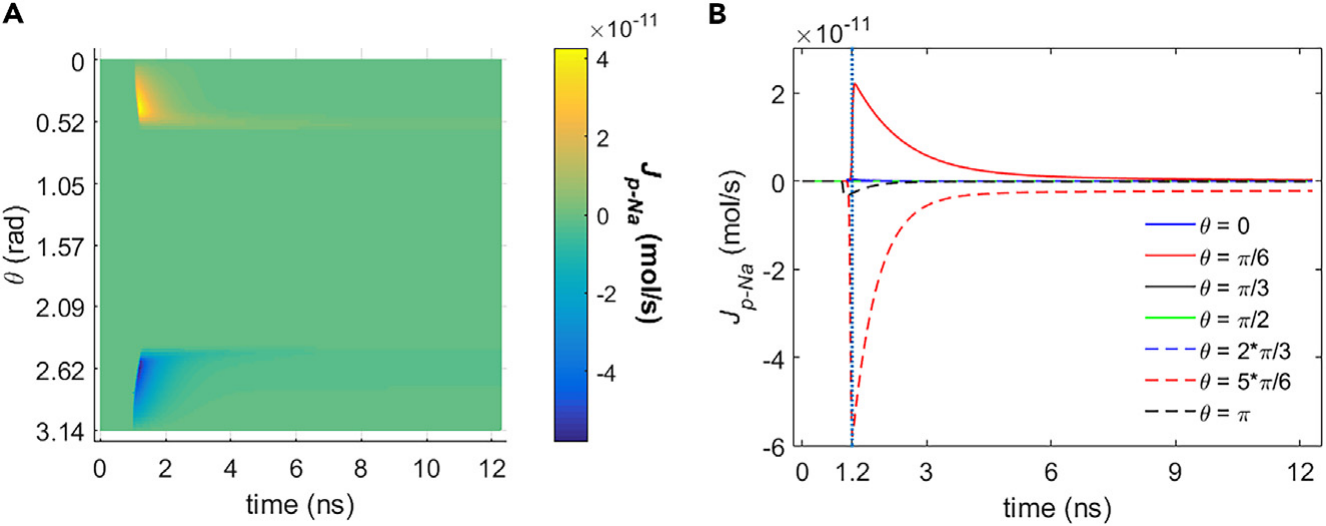
Transmembrane Na+ flow via hydrophilic pores of rat neostriatal neuron activated by 1.2 ns stimulation of 5 × 10^7^ V/m, 0.5 THz, 1 ps unipolar pulse train After the stimulation, the numerical calculations continue for around 11 ns. (A) The transmembrane Na+ flow with respect to polar angle ***θ*** versus time. (B) The transmembrane Na+ flow at ***θ*** = 0, ***π***/6, ***π***/3, ***π***/2, 2***π***/3, 5***π***/6, and ***π*** versus time.

**Figure 6:**
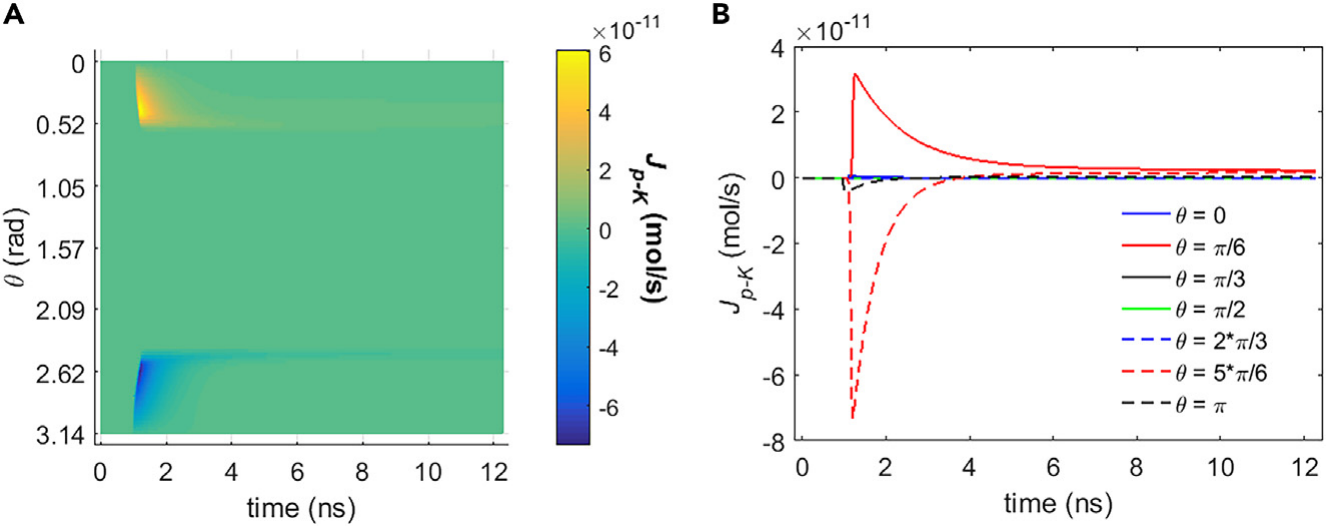
Transmembrane K+ flow via hydrophilic pores of rat neostriatal neuron activated by 1.2 ns stimulation of 5 × 10^7^ V/m, 0.5 THz, 1 ps unipolar pulse train After the stimulation, the numerical calculations continue for around 11 ns. (A) The transmembrane K+ flow with respect to polar angle ***θ*** versus time. (B) The transmembrane K+ flow at ***θ*** = 0, ***π***/6, ***π***/3, ***π***/2, 2***π***/3, 5***π***/6, and ***π*** versus time.

**Figure 7:**
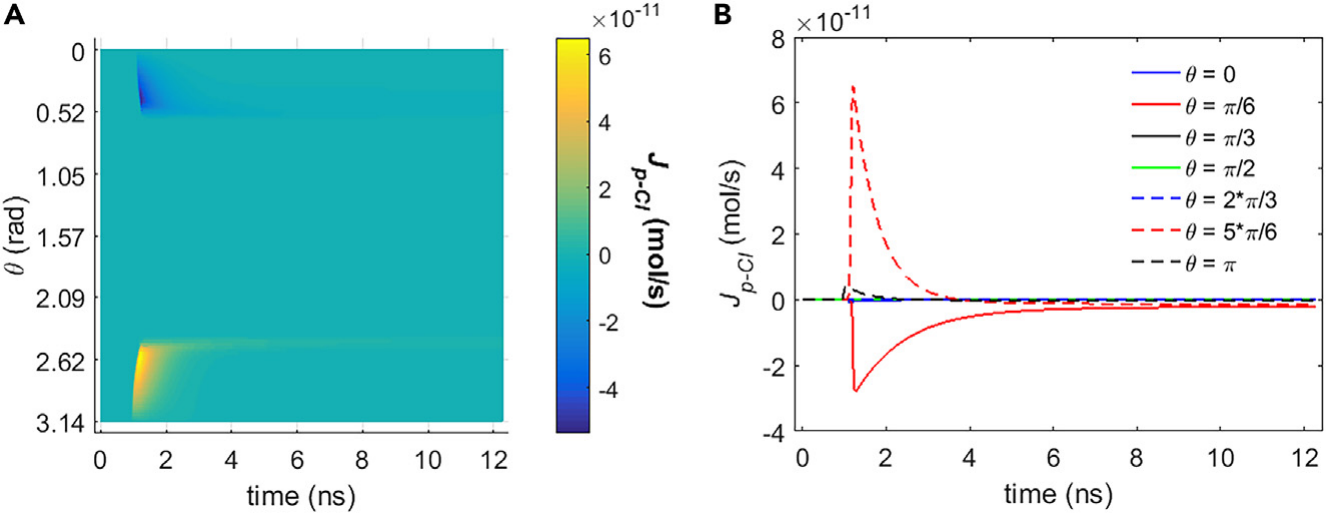
Transmembrane Cl− flow via hydrophilic pores of rat neostriatal neuron activated by 1.2 ns stimulation of 5 × 10^7^ V/m, 0.5 THz, 1 ps unipolar pulse train After the stimulation, the numerical calculations continue for around 11 ns. (A) The transmembrane Cl− flow with respect to polar angle ***θ*** versus time. (B) The transmembrane Cl− flow at ***θ*** = 0, ***π***/6, ***π***/3, ***π***/2, 2***π***/3, 5***π***/6, and ***π*** versus time.

**Figure 8:**
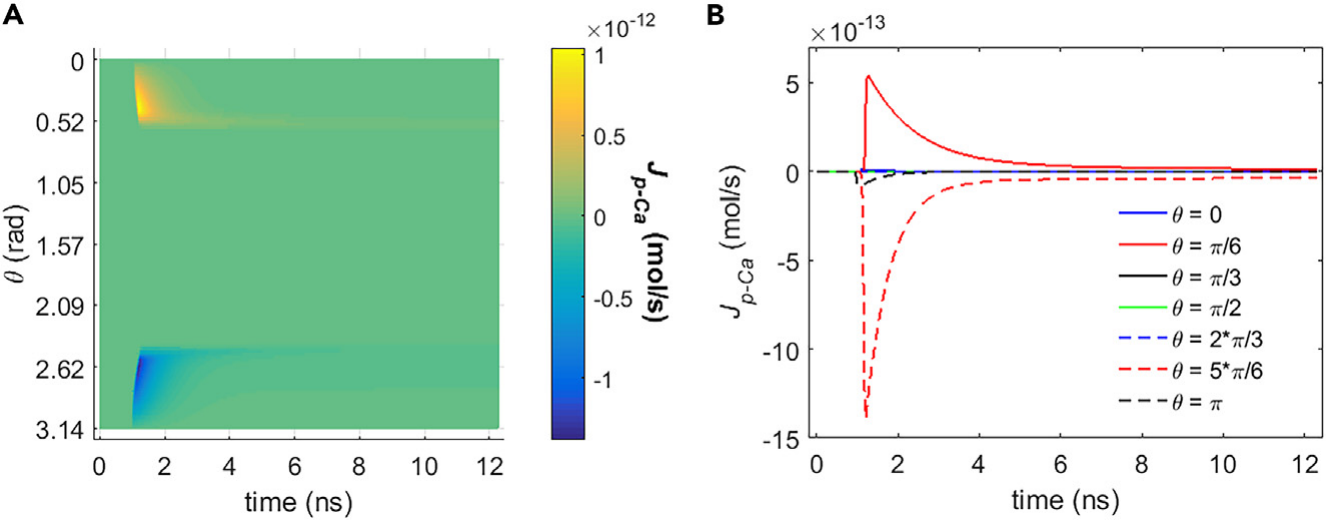
Transmembrane Ca2+ flow via hydrophilic pores of rat neostriatal neuron activated by 1.2 ns stimulation of 5 × 10^7^ V/m, 0.5 THz, 1 ps unipolar pulse train After the stimulation, the numerical calculations continue for around 11 ns. (A) The transmembrane Ca2+ flow with respect to polar angle ***θ*** versus time. (B) The transmembrane Ca2+ flow at ***θ*** = 0, ***π***/6, ***π***/3, ***π***/2, 2***π***/3, 5***π***/6, and ***π*** versus time.

**Figure 9:**
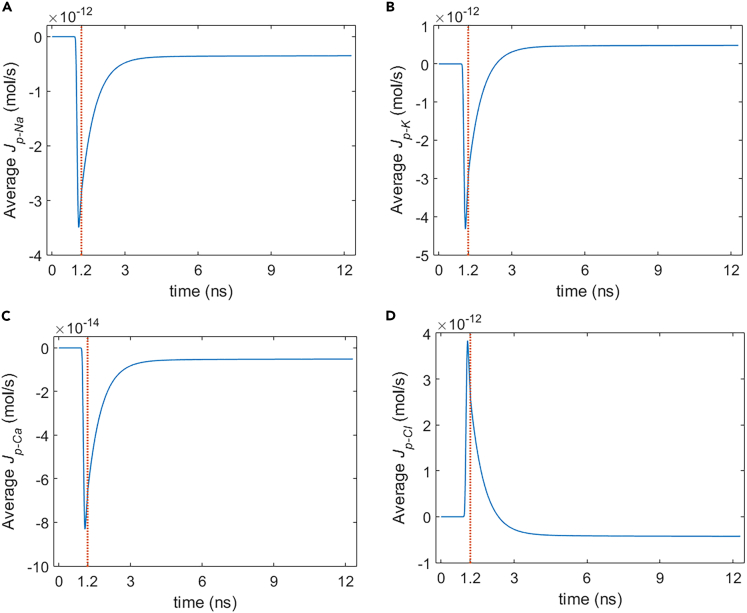
Average Na+, K+, Ca2+, and Cl− flow via hydrophilic pores in rat neostriatal neuron under the 1.2 ns stimulation of 5 × 10^7^ V/m, 0.5 THz, 1 ps unipolar pulse train After the stimulation, the numerical calculations continue for around 11 ns. (A–D) (A) Average Na+ flow, (B) average K+ flow, (C) average Ca2+ flow, (D) average Cl-flow.

It can be seen from Figures 5, 6, 7, and 8 that during the stimulation once the hydrophilic pores are activated at around 0.94 ns, the transmembrane ion flow of Na+, K+, Cl−, and Ca2+ via hydrophilic pores increases from zero and shortly reaches each maximum. After that, the life ion flow starts to decrease exponentially due to the decrease of membrane potential (Figure 3) when the hydrophilic pores in cell membrane get nearly stable (Figure 4). Figures 9A–9D are respectively the normal averages (nonweighted averages) with respect to the angular direction θ of Figures 5A, 6A, 7A, and 8A. From Figure 9, it can be seen that the average ion flows of Na+, K+, Cl−, and Ca2+ in a whole cell finally tend to nonzero values in around 11 ns after the stimulation.

**Figure 10:**
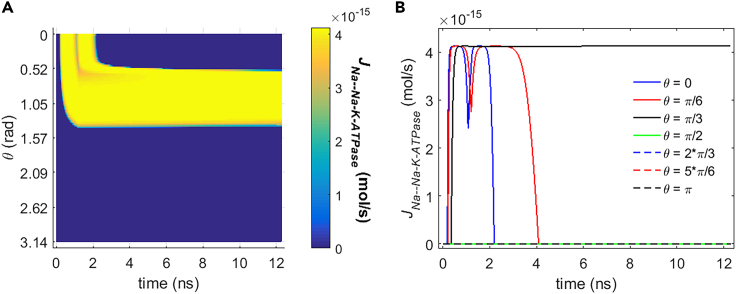
Transmembrane Na+ flow via Na+, K+-ATPase of rat neostriatal neuron activated by 1.2 ns stimulation of 5 × 10^7^ V/m, 0.5 THz, 1 ps unipolar pulse train After the stimulation, the numerical calculations continue for around 11 ns. (A) The transmembrane Na+ flow with respect to polar angle ***θ*** versus time. (B) The transmembrane Na+ flow at ***θ*** = 0, ***π***/6, ***π***/3, ***π***/2, 2***π***/3, 5***π***/6, and ***π*** versus time.

**Figure 11:**
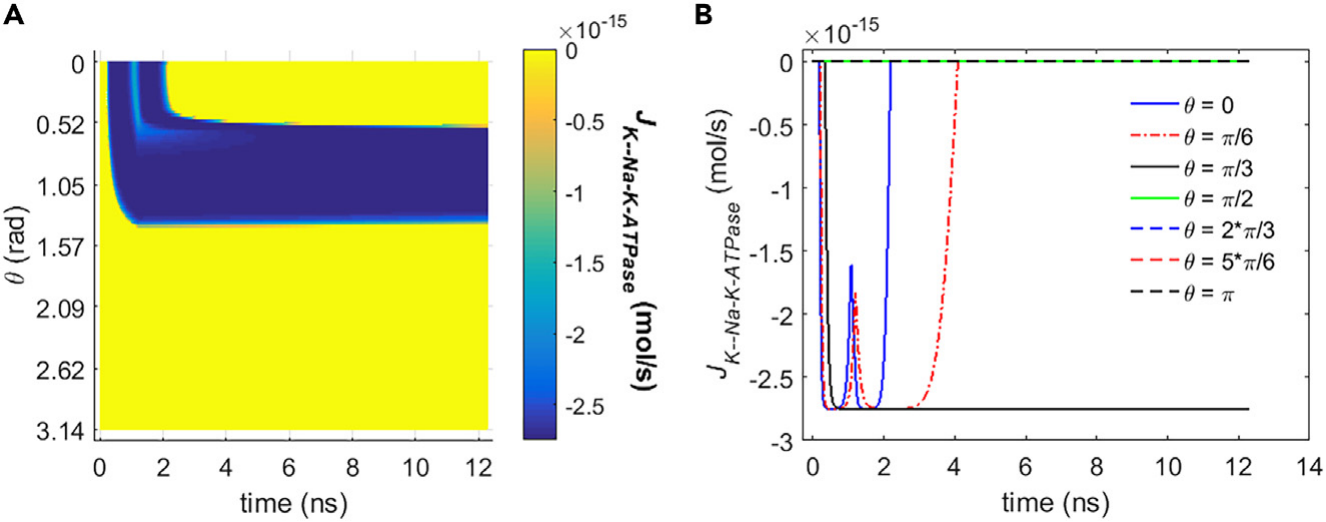
Transmembrane K+ flow via Na+, K+-ATPase of rat neostriatal neuron activated by 1.2 ns stimulation of 5 × 10^7^ V/m, 0.5 THz, 1 ps unipolar pulse train After the stimulation, the numerical calculations continue for around 11 ns. (A) The transmembrane K+ flow with respect to polar angle ***θ*** versus time. (B) The transmembrane K+ flow at ***θ*** = 0, ***π***/6, ***π***/3, ***π***/2, 2***π***/3, 5***π***/6, and ***π*** versus time.

Figures 10 and 11 show the transmembrane ion flows of Na+ and K+ via Na+, K+-ATPase under the stimulation. During the stimulation, the flows vary as a result of the variation of membrane potentials before the activation of hydrophilic pores (Figure 3). After the activation of hydrophilic pores during the stimulation, the variation of ion concentrations due to the ion flows via the pores also contributes to the ion flows via Na+, K+-ATPase. The Na+ and K+ flows via Na+, K+-ATPase are nonzero when the membrane potential increases from resting potential to the positive direction in the case of rat neostriatal neuron (Figure 2A). Thus, based on the variation of the membrane potential with time by the stimulation shown in Figure 3, the Na+ and K+ flows are nonzero in the range of θ from 0 to π/2 as shown in Figures 10 and 11. The Na+ and K+ flows increase first and then decrease with the increase of the membrane potential at θ = 0, π/6, and π/3; this is because according to the I-V response characteristics of Na+, K+-ATPase in rat neostriatal neuron shown in Figure 2A, the positive peaks of membrane potential at θ = 0 and π/6 are overlarge, and therefore the flows show an extraordinary opposite-direction-varying peak at θ = 0 and π/6 at around 1 ns in Figures 10 and 11. This reflects an inhibition effect on Na+, K+-ATPase functions, which is similar to the case of voltage-gated calcium channels.^12^ In contrast, because the peak is much smaller, it does not show an extraordinary opposite-direction-varying peak when the membrane potential is reaching toward its peak at θ = π/3. After reaching the peaks, the membrane potentials at θ = 0 and π/6 decrease steeply toward to nearly zero soon within 12 ns, so the flows have extraordinary varies first and then decrease to zero soon. In contrast, after reaching the peak, the membrane potential at θ = π/3 decreases comparably much slower and at 12 ns it still has a large magnitude, so the flows stay large and do not decrease to zero at θ = π/3. So the maximum flows in Figures 10B and 11B are always reached when θ is π/3.

**Figure 12:**
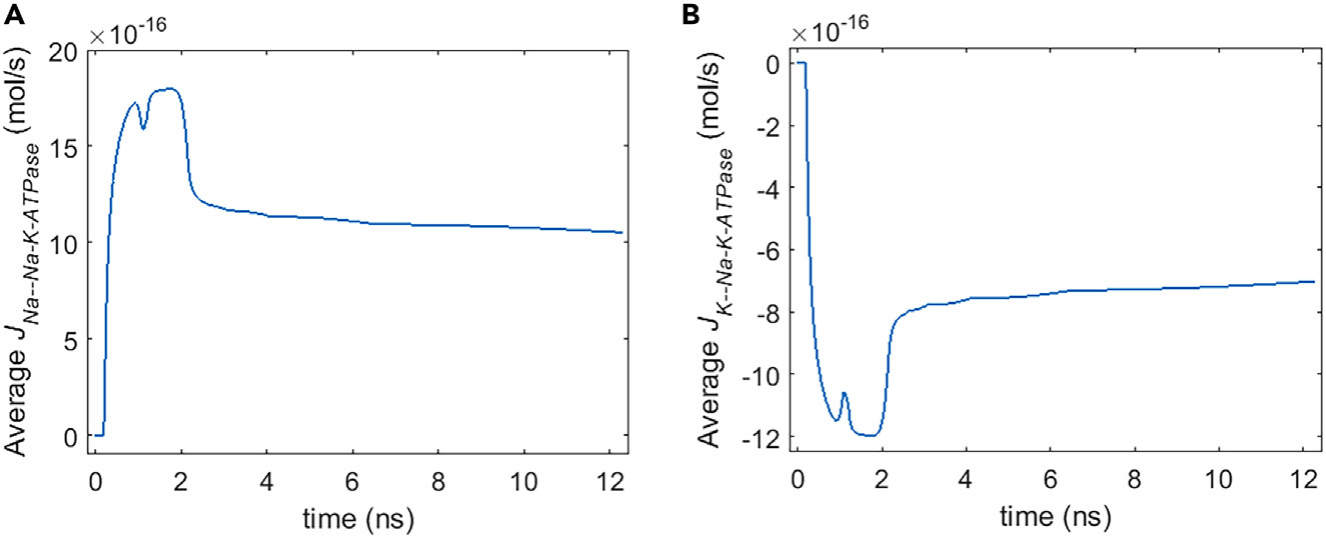
Average life ion flows via Na+, K+-ATPase of rat neostriatal neuron under the 1.2 ns stimulation of 5 × 10^7^ V/m, 0.5 THz, 1 ps unipolar pulse train After the stimulation, the numerical calculations continue for around 11 ns. (A and B) (A) Average Na+ flow, (B) average K+ flow.

By comparing Figures 10 and 11 and Figures 5 and 6, it can be seen that the life ion flows via Na+, K+-ATPase are nearly four orders of magnitude smaller than those via hydrophilic pores during the stimulation. After the stimulation, because the hydrophilic pores stay in the cell membrane, the ion flows via Na+, K+-ATPase are still much smaller than those via hydrophilic pores. The Na+ flow has positive values, and K+ flow is negative in Figures 10 and 11, which means that Na+ ions are transported from the cell into extracellular environment and that K+ ions are transported from the extracellular environment into the cell by Na+, K+-ATPase. Those are in accordance with the reality. Figures 12A and 12B are respectively the normal averages (nonweighted averages) with respect to the angular direction θ of Figures 10A and 11A. From Figure 12, it can be seen that the variation of the average life ion flows via Na+, K+-ATPase tends to be gentle in around 11 ns after the stimulation.

#### Variation of life ion concentrations in cell

**Figure 13:**
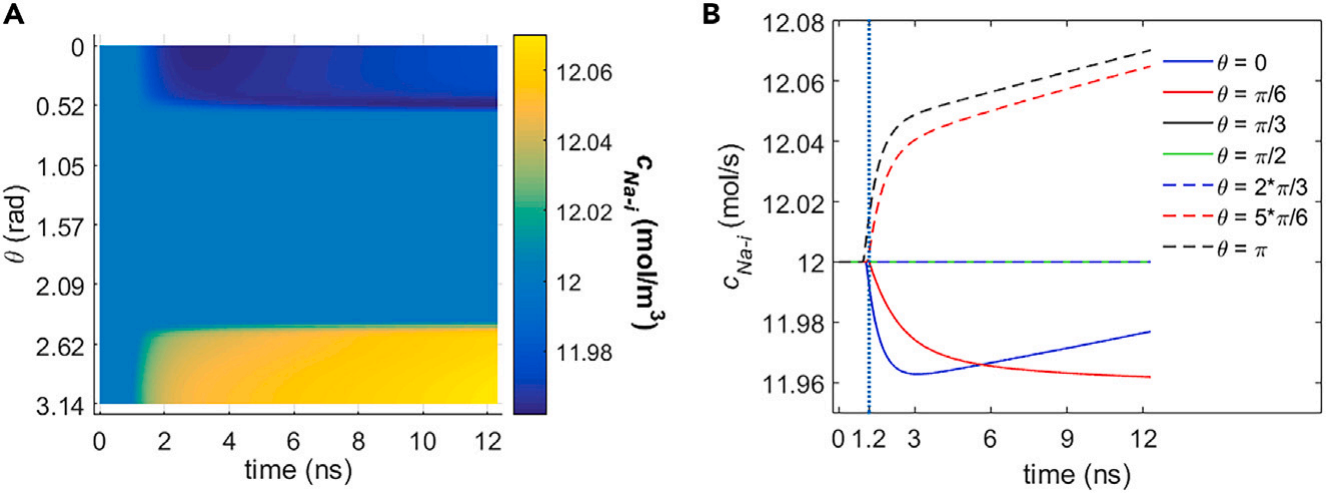
Variation of intracellular Na+ concentration due to the transmembrane Na+ flow via hydrophilic pores and Na+, K+-ATPase in rat neostriatal neuron under the 1.2 ns stimulation of 5 × 10^7^ V/m, 0.5 THz, 1 ps unipolar pulse train After the stimulation, the numerical calculations continue for around 11 ns. (A) Variation of intracellular Na+ concentration with respect to polar angle ***θ*** versus time. (B) Variation of intracellular Na+ concentration at ***θ*** = 0, ***π***/6, ***π***/3, ***π***/2, 2***π***/3, 5***π***/6, and ***π*** versus time.

**Figure 14:**
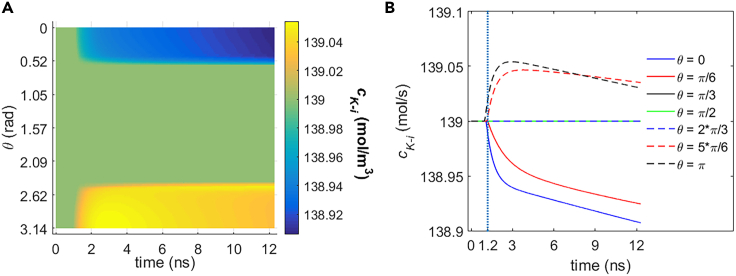
Variation of intracellular K+ concentration due to the transmembrane K+ flow via hydrophilic pores and Na+, K+-ATPase in rat neostriatal neuron under the 1.2 ns stimulation of 5 × 10^7^ V/m, 0.5 THz, 1 ps unipolar pulse train After the stimulation, the numerical calculations continue for around 11 ns. (A) Variation of intracellular K+ concentration with respect to polar angle ***θ*** versus time. (B) Variation of intracellular K+ concentration at ***θ*** = 0, ***π***/6, ***π***/3, ***π***/2, 2***π***/3, 5***π***/6, and ***π*** versus time.

Figures 13 and 14 show the variation of intracellular Na+ and K+ ion concentration at different polar angles θ due to the ion flows via the cell membrane hydrophilic pores and Na+, K+-ATPase under the 1.2 ns stimulation of 5 × 10^7^ V/m, 0.5 THz, 1 ps unipolar pulse train. From the figures, the intracellular ion concentrations of Na+ and K+ both decrease near θ = 0 and increase near θ = π during the stimulation. The reason is as follows. Because the ion flow component due to the electric field across the cell membrane is far larger than the component due to the concentration difference across the membrane in the case of the ion flows via hydrophilic pores,^19^ the variation of the concentration at different polar angles θ is mainly because of the ion flow component due to the electric field. Furthermore, the direction of the stimulated THz electric field is pointing to extracellular environment near θ = 0 and intracellular environment near θ = π (see Figure 1). And Na+ and K+ ions are both positive charges. Thus, during the stimulation, Na+ and K+ ions are both transported from the cell to extracellular environment near θ = 0 and from the extracellular environment to the cell near θ = π, leading to the corresponding variation of the ion concentrations of Na+ and K+. And the trend of the variation of the ion concentrations at different θ keeps for a short time after the stimulation (Figures 13 and 14); this is because during this time the membrane potentials are still large enough (Figure 3).

At the relatively long time after the stimulation, the Na+ concentrations near θ = 0 and π both start to increase, and the K+ concentration near θ = 0 and π both start to decrease; this is because at this time the membrane potentials are close to 0 (Figure 3), and then the ion flow component due to the electric field becomes negligible compared with that due to the concentration difference. Thus, the variation of the concentration at different θ is mainly because of the ion flow component due to the concentration difference across the cell membrane. It is worth mentioning that there is an exception at θ = π/6 in the case of Na+ (Figure 13B), and this is because the membrane potential is not close to 0 at this moment so the ion flow component due to the electric field at θ = π/6 is still large and nonnegligible.

**Figure 15:**
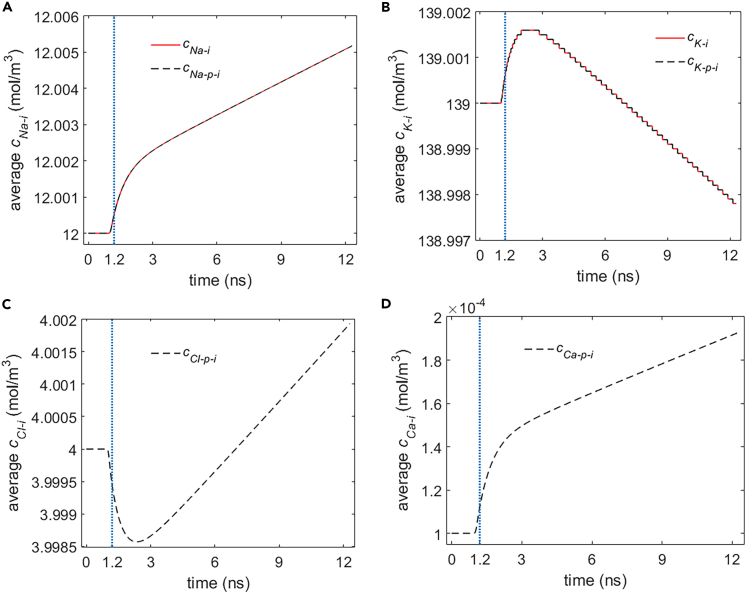
Variation of average intracellular ion concentrations of Na+ c_Na__-i_ and K+ c_K-i_ due to the transmembrane life ion flow via hydrophilic pores and Na+, K+-ATPase in rat neostriatal neuron under the 1.2 ns stimulation of 5 × 10^7^ V/m, 0.5 THz, 1 ps unipolar pulse train (A–D) The c_Na-p-i_, c_K-p-i_, c_Cl-p-i_, and c_Ca-p-i_ are respectively the Na+, K+, Cl−, and Ca2+ concentration due to the ion flow via exclusive hydrophilic pores. After the stimulation, the numerical calculations continue for around 11 ns. (A) c_Na-i_ and c_Na-p-i_, (B) c_K-i_ and c_K-p-i_, (C) c_Cl-p-i_, (D) c_Ca-p-i_.

Figure 15 shows the variation of the average ion concentrations of Na+, K+, Ca2+, and Cl− in cell under the stimulation. It can be seen that there are abnormal variation rates or tendencies at the time during and shortly after the stimulation (before around 3 ns in Figure 15) for each type of the ions; this is because of the effect of the ion flow component due to the electric field across the cell membrane on the transmembrane ion flow.^19^ And at a relatively long time after the stimulation, the variation tendency for each type of the ions is compliant with the tendency due to their concentration differences across the cell membrane. This is because the ion flow component due to the electric field becomes negligible at this time in comparison to that due to the concentration difference.

It should be noted that the variations of the intracellular ion concentrations for Na+, K+, and Cl− seem to be trivial compared with their large basal ion concentration. And owing to the tiny basal ion concentration in cell for Ca2+, the variation of intracellular Ca2+ concentration is nonnegligible, nearly two times the basal ion concentration in around 11 ns after the stimulation. It should also be noted that the effect of Na+, K+-ATPase on the Na+ and K+ ion concentration in cell during the life ion flow via hydrophilic pores is negligible (Figures 15A and 15B). This is consistent with the fact that the life ion flows via Na+, K+-ATPase are much smaller than the flows via hydrophilic pores (Section life ion flows via Na+, K+-ATPase and hydrophilic pores).

#### Na+, K+-ATPase power dissipation

**Figure 16:**
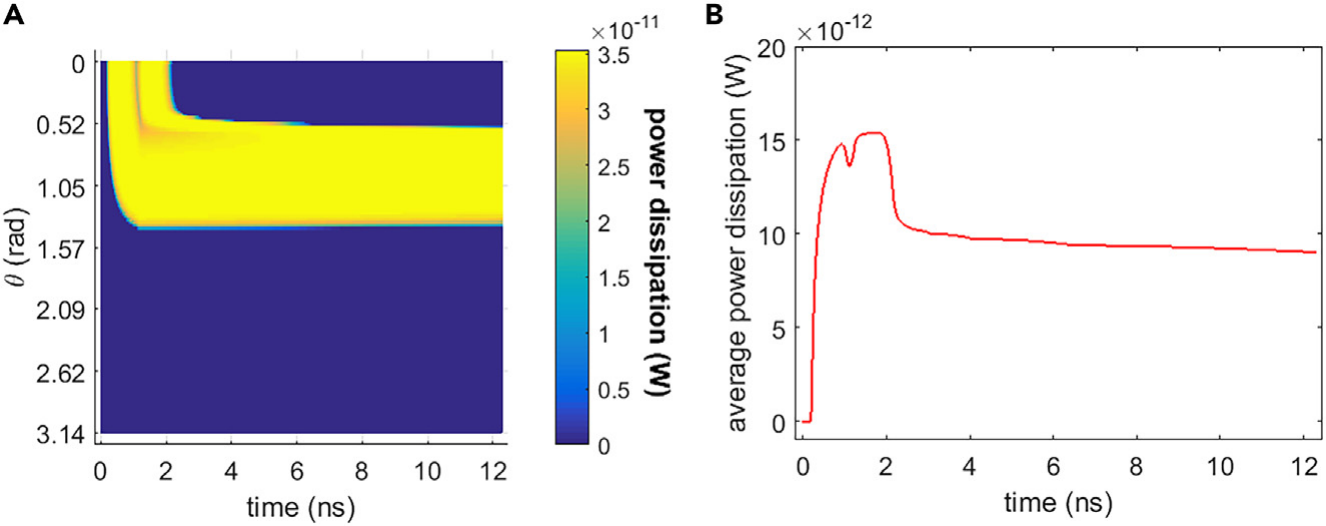
Power dissipation of Na+, K+-ATPase during the transmembrane life ion flow in rat neostriatal neuron under the 1.2 ns stimulation of 5 × 10^7^ V/m, 0.5 THz, 1 ps unipolar pulse train After the stimulation, the numerical calculations continue for around 11 ns. (A) The power dissipation with respect to polar angle ***θ*** versus time. (B) The power dissipation at ***θ*** = 0, ***π***/6, ***π***/3, ***π***/2, 2***π***/3, 5***π***/6, and ***π*** versus time.

Figure 16 shows the power dissipation of Na+, K+-ATPase during the life ion flows via hydrophilic pores under the stimulation. It can be seen that the power dissipation varies a lot during and shortly after the stimulation (before around 3 ns) when the membrane potentials vary drastically. And the variation of the power dissipation tends to be gentle at a relatively long time after the stimulation. It is clear that the average power dissipation is as low as around the level of 10^−11^ W. However, it is qualitatively several orders of magnitude larger than the power dissipation that is at the level of 10^−18^ W in the ion flow via protein Ca2+ channels.^20^ The reasons are as follows: the life ion flow via hydrophilic pores in Figures 5, 6, 7, and 8 is far larger than the ion flow via voltage-gated Ca2+ channels (VGCCs) as seen in literature.^14^ Thus, the variations of membrane potentials and intracellular ion concentrations are more drastic due to ion flow via hydrophilic pores seen in Figures 3 and 15 than due to ion flow via VGCCs as seen in literature^14^; this leads to a much larger ion flow via the ion ATPase in the case of hydrophilic pores compared with that in the case of VGCCs. As a result, the power dissipation in the case of ion flow via hydrophilic pores is much larger than that in the case of ion flow via the VGCCs.

#### Comparison in different cell types

**Figure 17:**
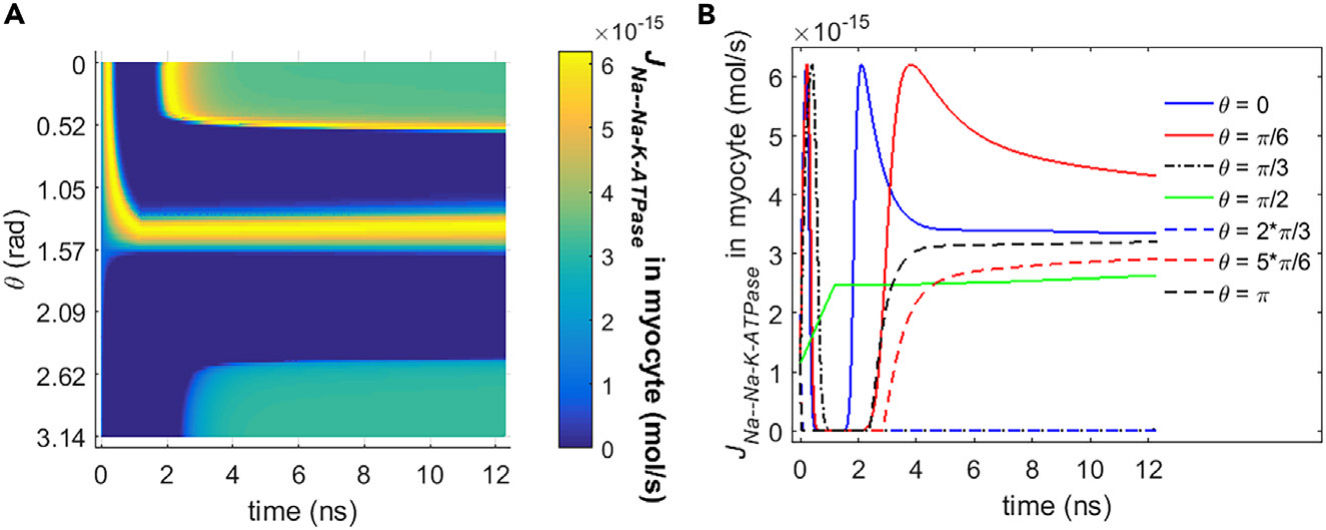
Transmembrane Na+ flow via Na+, K+-ATPase of guinea pig ventricular myocyte activated by 1.2 ns stimulation of 5 × 10^7^ V/m, 0.5 THz, 1 ps unipolar pulse train After the stimulation, the numerical calculations continue for around 11 ns. (A) The transmembrane Na+ flow with respect to polar angle ***θ*** versus time. (B) The transmembrane Na+ flow at ***θ*** = 0, ***π***/6, ***π***/3, ***π***/2, 2***π***/3, 5***π***/6, and ***π*** versus time.

**Figure 18:**
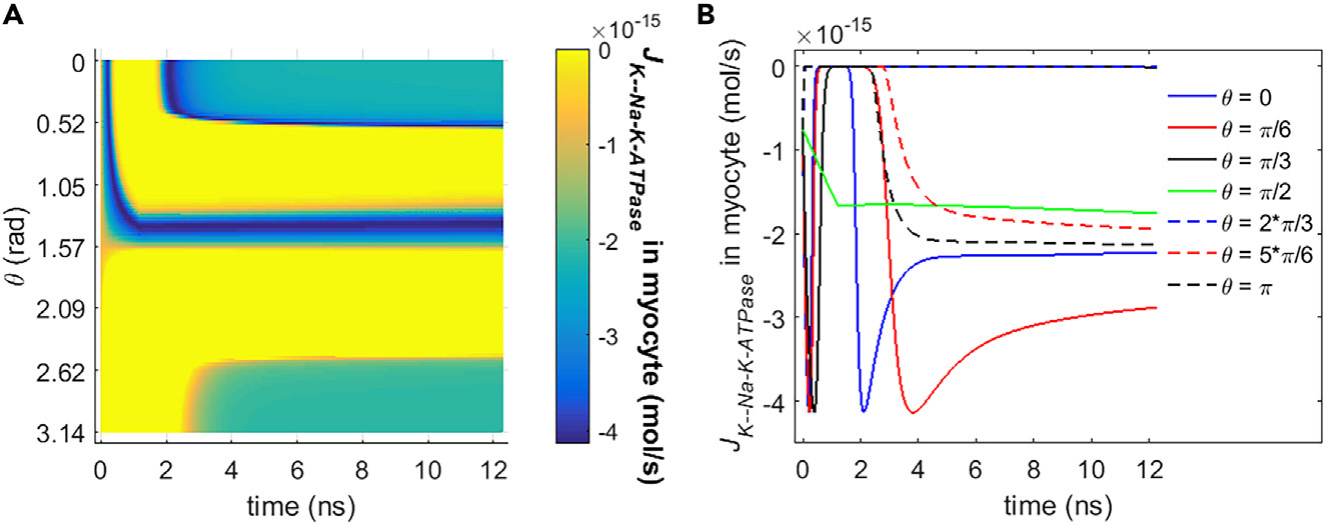
Transmembrane K+ flow via Na+, K+-ATPase of guinea pig ventricular myocyte activated by 1.2 ns stimulation of 5 × 10^7^ V/m, 0.5 THz, 1 ps unipolar pulse train After the stimulation, the numerical calculations continue for around 11 ns. (A) The transmembrane K+ flow with respect to polar angle ***θ*** versus time. (B) The transmembrane K+ flow at ***θ*** = 0, ***π***/6, ***π***/3, ***π***/2, 2***π***/3, 5***π***/6, and ***π*** versus time.

**Figure 19:**
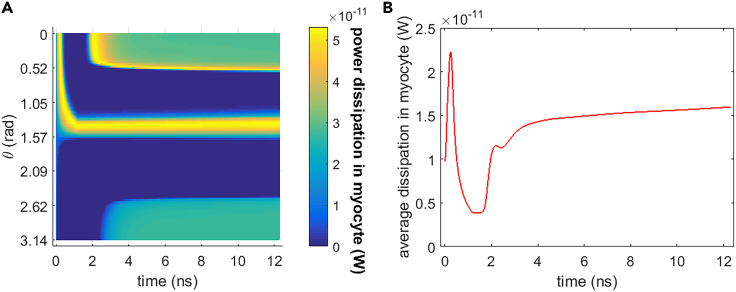
Power dissipation of Na+, K+-ATPase during the transmembrane life ion flow in guinea pig ventricular myocyte under the 1.2 ns stimulation of 5 × 10^7^ V/m, 0.5 THz, 1 ps unipolar pulse train After the stimulation, the numerical calculations continue for around 11 ns. (A) The power dissipation with respect to polar angle ***θ*** versus time. (B) The power dissipation at ***θ*** = 0, ***π***/6, ***π***/3, ***π***/2, 2***π***/3, 5***π***/6, and ***π*** versus time.

In addition to the rat neostriatal neuron, the Na+ and K+ flow and the power dissipation of Na+, K+-ATPase during the life ion flow via hydrophilic pores are investigated in another cell type, guinea pig ventricular myocyte. Because the changes in ion concentrations are nearly negligible for Na+ and K+ ions (see Figure 15), the variations of the ion flow via Na+, K+-ATPase are mainly determined by the variations of membrane potentials. As the membrane potentials continue to increase toward their peaks (see Figure 3), the ion flows reach their maximums more shortly and then decrease more shortly even to zero in the case of guinea pig ventricular myocyte (Figures 17 and 18) compared with the case of rat neostriatal neuron (Figures 10 and 11). This is because the I-V characteristics in guinea pig ventricular myocyte has a narrower shape compared with that in rat neostriatal neuron (see Figure 2). Thus, the increase and the decrease vary more shortly in the case of guinea pig ventricular myocyte than in the case of rat neostriatal neuron. Besides, the ion flows at most of the θ do not decrease to zero at the time from 4 ns to around 12 ns compared with the rat neostriatal neuron. This is because the I-V curve has a shift toward more negative membrane potential compared with that in rat neostriatal neuron (see Figure 2). Thus, at those membrane potentials at the time from 4 ns to around 12 ns, the ion flows in guinea pig ventricular myocyte are nonzero. The difference of ion flows in these two different cell types leads to the difference of power dissipation of Na+, K+-ATPase between Figures 16 and 19 according to Equation 21.

From Figures 17, 18, and 19, it can be seen that the Na+ flow, K+ flow, and power dissipation in the case of guinea pig ventricular myocyte vary a lot during and shortly after the stimulation when the membrane potentials vary drastically. And at a relatively long time after the stimulation, the variations of ion flows and power dissipation tend to be gentle. Those are the same as the case of rat neostriatal neuron. Besides, the average power dissipation of Na+, K+-ATPase in guinea pig ventricular myocyte has the similar order of magnitude as that in rat neostriatal neuron, namely, the level of 10^−11^ W, although the specific values are different. As a result, the Na+, K+-ATPase in rat neostriatal neuron and guinea pig ventricular myocyte behaves similar during the life ion flow via hydrophilic pores under the stimulation.

### Effect of the frequency of the stimulation

#### Effect of the frequency on the variation quantity of ion concentration

**Figure 20:**
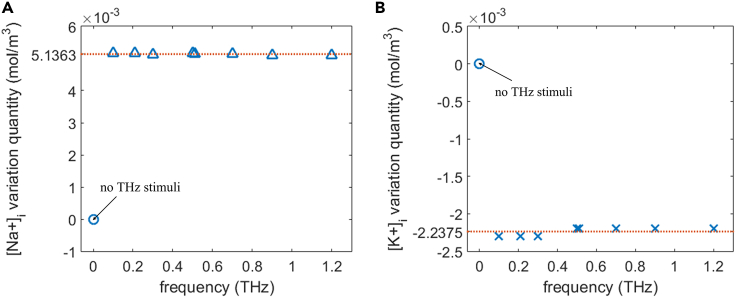
Variation quantity of ion concentration induced by the flow via hydrophilic pores and Na+, K+-ATPase versus frequency with and without the stimulation (A and B) Variation quantity of intracellular ion concentration due to the life ion flow via cell membrane hydrophilic pores and Na+, K+-ATPase at around 12.3 ns with respect to different stimulation frequencies under the 1.2 ns stimulation of 5 × 10^7^ V/m, 1 ps unipolar pulse train, the variation quantity without the THz stimulation is also plotted (“no THz stimuli”) in each figure: (A) variation quantity of intracellular Na+ concentration [Na+]i versus frequency, (B) variation quantity of intracellular K+ concentration [K+]i versus frequency.

Figure 20 shows the variation quantities of intracellular Na+ and K+ concentrations as a result of life ion flow via hydrophilic pores and Na+, K+-ATPase with respect to frequency in 0.1–1.2 THz. In Figure 20, the variation quantity without the THz stimulation is also plotted. The cell is under 1.2 ns stimulation of 5 × 10^7^ V/m, 1 ps unipolar pulse train, and the variation quantities are accumulated at 12.3 ns. It is apparent that the variation quantities of the Na+ and K+ ion concentrations are both nearly invariant with respect to the frequency. And the effect of the THz stimulation is obvious by comparing the variation quantities with and without the stimulation.

#### Effect of the frequency on the power dissipation

**Figure 21:**
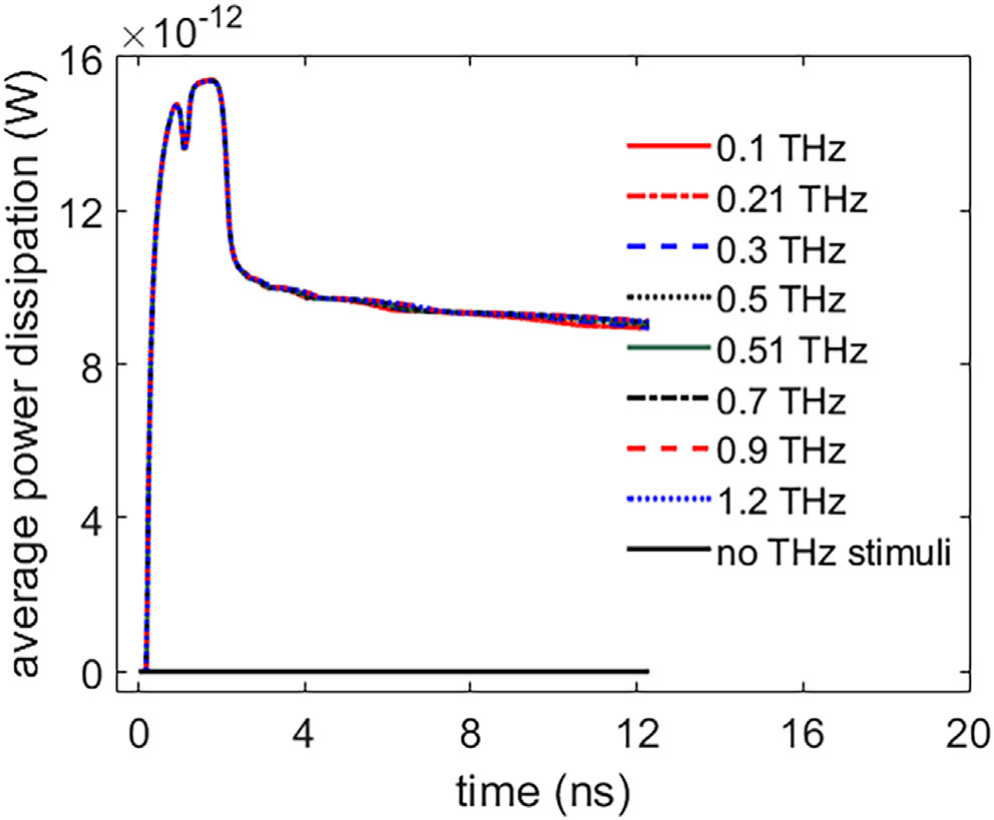
Average power dissipation of Na+, K+-ATPase during the life ion flow via hydrophilic pores with respect to time at different stimulation frequencies 0.1, 0.21, 0.3, 0.5, 0.51, 0.7, 0.9, and 1.2 THz under the 1.2 ns stimulation of 5 × 10^7^ V/m, 1 ps unipolar pulse train and the average power dissipation without the THz stimulation (“no THz stimuli”)

Figure 21 shows the average power dissipation of Na+, K+-ATPase with time during the life ion flow via hydrophilic pores at different frequencies. It can be seen from the figure that the variation of the power dissipation is nearly the same under different frequencies in 0.1–1.2 THz. And the effect of the THz stimulation on the power dissipation is significant by comparing with that of no THz stimuli. The activation of Na+, K+-ATPase and hydrophilic pores in the cell is mainly due to the increase of the absolute values of membrane potentials under terahertz unipolar picosecond pulse train stimulation in the range of 0.1–1.2 THz. Because the duty cycles of the pulse trains are the same at different frequencies, the integrals of the electric fields delivered to the cell in terms of time are the same. Therefore, the frequency of the terahertz stimulation has no impact on the life ion flows, the variation quantities of ion concentrations accumulated, and the power dissipation in this narrow frequency range. As a result, the results in Section cell life ion flow and Na+, K+-ATPase power dissipation under the stimulation keep tenable under different stimulation frequencies in 0.1–1.2 THz.

### Close of Na+, K+-ATPase

Section cell life ion flow and Na+, K+-ATPase power dissipation under the stimulation shows that Na+, K+-ATPase does not close at around 11 ns after the stimulation. In order to investigate whether Na+, K+-ATPase closes during the life ion flow via hydrophilic pores, a longer time around 80 ns simulation is conducted. And meanwhile the stimulation duration is reduced from 1.2 ns to 1.1 ns to increase the ratio of the time after the stimulation to the time during the stimulation.

**Figure 22:**
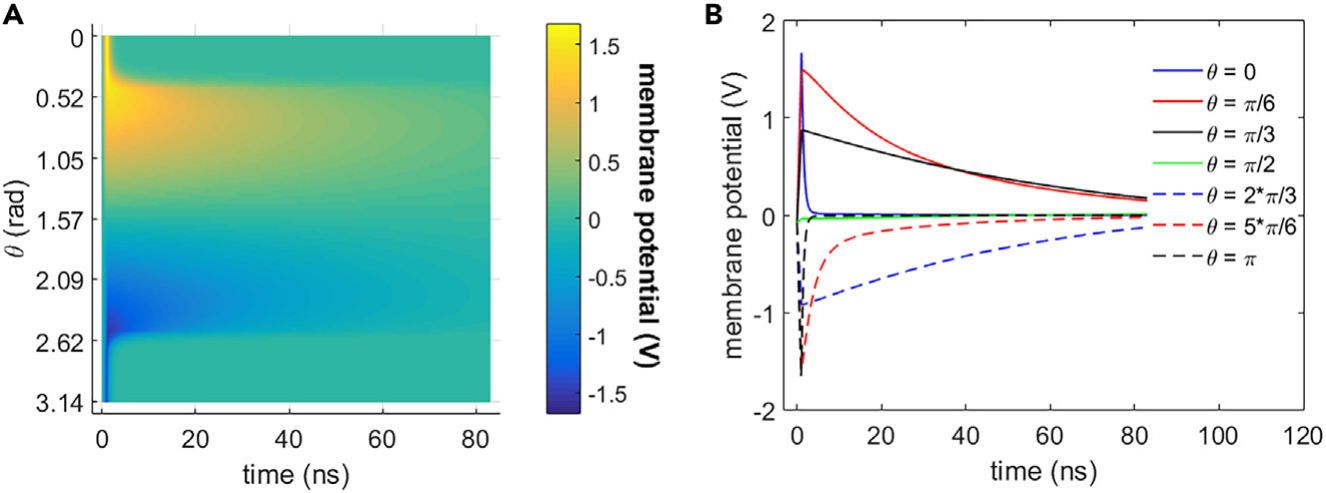
Membrane potential of rat neostriatal neuron versus time under the 1.1 ns stimulation of 5 × 10^7^ V/m, 0.5 THz, 1 ps unipolar pulse train After the stimulation, the numerical calculations continue for around 80 ns. (A) Membrane potential with respect to polar angle ***θ*** versus time. (B) Membrane potential at ***θ*** = 0, ***π***/6, ***π***/3, ***π***/2, 2***π***/3, 5***π***/6, and ***π*** versus time.

**Figure 23:**
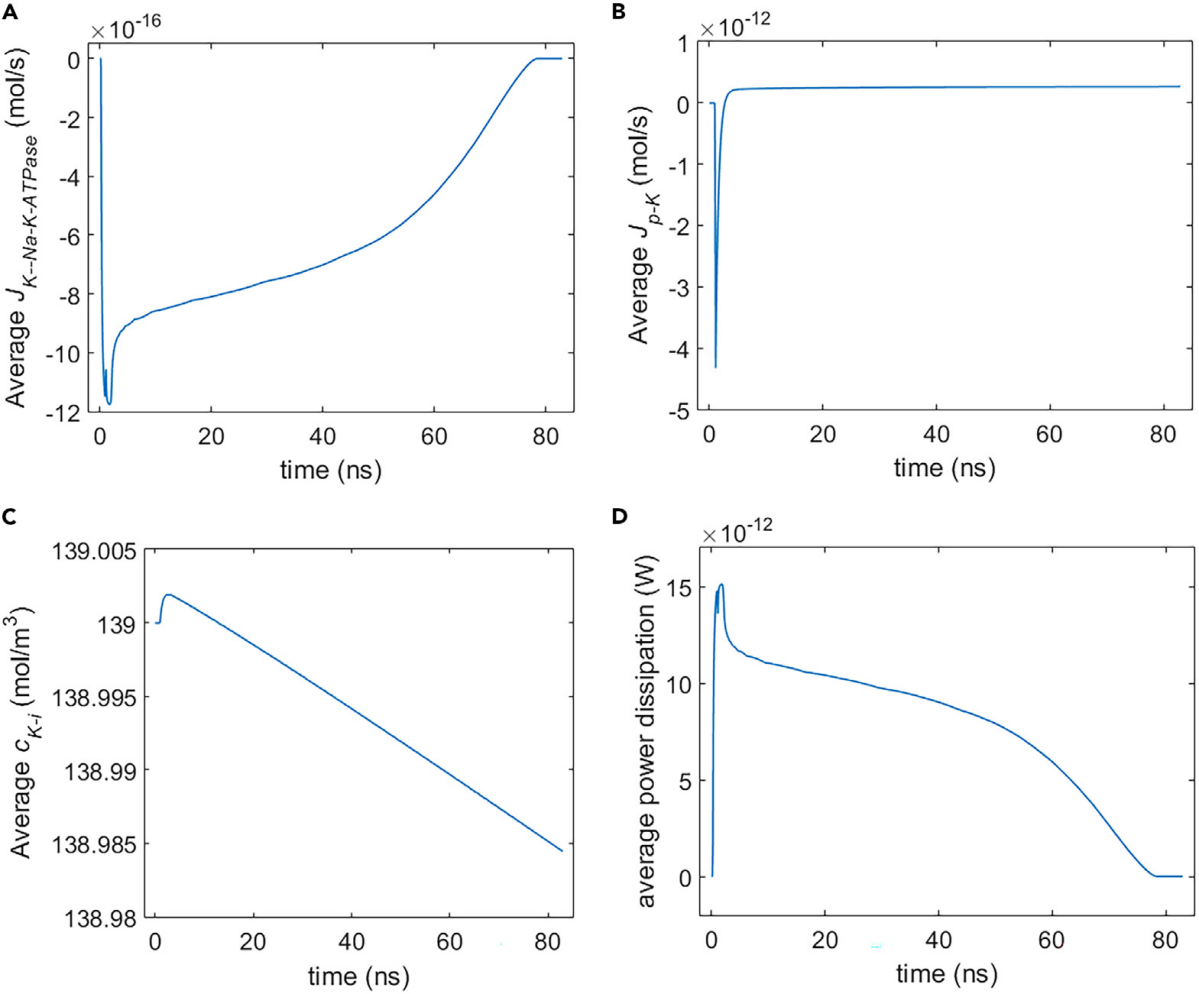
Life ion flow, ion concentration, and power dissipation in rat neostriatal neuron in around 80 ns after the 1.1 ns stimulation of 5 × 10^7^ V/m, 0.5 THz, 1 ps unipolar pulse train (A) Average K+ flow via Na+, K+-ATPase. (B) Average K+ flow via hydrophilic pores. (C) Variation of average intracellular K+ concentration due to the flow via hydrophilic pores and Na+, K+-ATPase. (D) Average power dissipation of Na+, K+-ATPase during the life ion flow via hydrophilic pores.

As we can see from Figures 22 and 23, the membrane potential of the cell keeps approaching nearly to zero, and the life ion flow via hydrophilic pores tends to be stable after 11 ns after the 1.1 ns stimulation of 5 × 10^7^ V/m, 0.5 THz, 1 ps unipolar pulse train. After a drastic variation during and shortly after the stimulation, the flow via Na+, K+-ATPase tends to be gentle and slowly decreases to zero with time mainly due to the decrease of membrane potentials. Na+, K+-ATPase closes at around 80 ns.

Besides, from Figure 23, the intracellular K+ concentration keeps decreasing slowly in a relatively long time after the stimulation, because the flow via Na+, K+-ATPase is far smaller than that via hydrophilic pores. Nevertheless, the change in intracellular K+ concentration keeps negligible at around 80 ns after the stimulation. The power dissipation of Na+, K+-ATPase is at the level of 10^−11^ W, and it decreases to zero when Na+, K+-ATPase closes.

## Discussion

The activation of hydrophilic pores by THz unipolar picosecond pulse train stimulation causes enormous transmembrane life ion flows. Those ion flows are able to be far larger than the ion flow via protein ion channels and thus mask the other ion flows. The prevail of ion flow via hydrophilic pores leads to a drastic change in membrane potential, that is, a significant increase followed by a relatively slower but persistent decrease to approximately zero. In comparison, in the case where hydrophilic pores are not being activated, the change in membrane potential has much smaller amplitude.^12^^,^^14^^,^^20^ The membrane potentials vibrate with terahertz frequency around resting potential.^12^^,^^14^^,^^20^ Those lead to the fact that the ion flow via the ion ATPase with activation of hydrophilic pores is far larger than the flow via the ATPase without the activation of hydrophilic pores. Those lead to a tremendous energy consumption with the activation of hydrophilic pores compared with the case where hydrophilic pores are not activated. Besides, the hydrophilic pores show no signs of turning off for around 80 ns after the 1.1 ns stimulation. Then, the ion flow via the pores stays so the membrane potential keeps nearly zero instead of being back to resting potential for at least 80 ns. In this case, the cell physiological functions^21^^,^^22^^,^^23^ might be essential to make the hydrophilic pores closed before the cell is able to respond to the next stimulation. Hence, some time is necessary for the restoration of the cell back to resting conditions when it is ready for another stimulation or information communication. As a result, the THz unipolar picosecond pulse train stimulation might serve as an end signal of last communication or a reset signal for next communication in cells.

### Conclusions

Cell membrane Na+, K+-ATPases are activated during the life ion flows via cell membrane hydrophilic pores under the terahertz unipolar picosecond pulse train stimulation. After the stimulation, the Na+, K+-ATPase and hydrophilic pores can stay open for tens of nanoseconds before the close of Na+, K+-ATPase. The life ion flow via Na+, K+-ATPase is several orders of magnitude smaller than the flow via hydrophilic pores and has opposite transmembrane transport direction to the flow via the pores at a relatively long time after the stimulation. The life ion flow via the pores and Na+, K+-ATPase causes a negligible change in intracellular Na+ and K+ concentration even at a relatively long time 80 ns after the stimulation when the Na+, K+-ATPase closes. In two different types of cells, rat neostriatal neuron and guinea pig ventricular myocyte, the power dissipations of Na+, K+-ATPase during the life ion flow via hydrophilic pores are both at as low as around the level of 10^−11^ W. And the power dissipation becomes zero after the close of Na+, K+-ATPase. This level of power dissipation is still qualitatively far larger than the power dissipation (the level of 10^−18^ W) of cell membrane Ca2+ ATPase during the flow via voltage-gated calcium channels under terahertz bipolar picosecond pulse train stimulation where no hydrophilic pore is formed.^20^ This might indicate that the activation of hydrophilic pores may cause a drastic increase in energy consumption of cell metabolic energy during the information communication of cells. The results also show that the conclusions are tenable under different stimulation frequencies in 0.1–1.2 THz. The theoretical studies lay the foundations for the research on the information communication mechanisms in the cell under terahertz stimulation.

### Limitations of the study

This investigation is based on the interaction theory between terahertz fields and ions at the cellular level,^5^^,^^14^ so the stimulation frequency is confined in the low-frequency range of THz range in order to satisfy that the wavelength of the THz stimulation in the cellular environment is far larger than the cell system size as mentioned in Section cell model of ion flow by the stimulation in method details in STAR methods. Besides, for the frequency lower than 0.1 THz, which is beyond the scope of the terahertz frequency range, there might be some unknown microwave effects that are not taken into considerations in the model. Thus, current model is unsuitable for the investigations in the case that the frequency is below THz range. As a consequence, this investigation is focused on the stimulation of the narrow THz frequency band in 0.1–1.2 THz.

It is worth mentioning that many protein functions are associated with or governed by the natural vibration motions in proteins at sub-THz frequencies in solution as investigated in the literatures.^24^^,^^25^^,^^26^^,^^27^ Then, under the terahertz stimulation, the interaction of the electromagnetic stimulation at sub-THz frequencies and the natural vibration motions in protein molecules would have impact on the protein functions and thus lead to a potential amplification/deamplification of the protein activity. This, in the case of the ion channel protein in the cell membrane, is probably the amplification/deamplification of ion flow via the ion channel protein. Thus, the evaluation of the life ion flows via Na+, K+-ATPase by the stimulation might be overestimated or underestimated a few.

## STAR★Methods

### Key resources table

**Table:** 

REAGENT or RESOURCE	SOURCE	IDENTIFIER
**Deposited data**		

data of Na+, K + -ATPase energy consumption in ion flow of hydrophilic pores by terahertz unipolar stimulation	This paper	https://zenodo.org/record/8252966

**Software and algorithms**		

C codes for numerical calculation program in this study	This paper	https://zenodo.org/record/8252966
MATLAB codes for results plotting	This paper	https://zenodo.org/record/8252966

### Resource availability

#### Lead contact

Further information and requests for resources should be directed to and will be fulfilled by the lead contact, Wenfei Bo (bowf@foxmail.com/bowf@nudt.edu.cn).

#### Materials availability

This study did not generate new unique reagents.

### Experimental model and study participant details

The experimental data of rat neostriatal neuron^28^ and guinea pig ventricular myocyte^29^ were used in the study. As listed in the literature,^28^ single neostriatal neurons were dissociated from 13 to 19-day-old Wistar rats with both sexes and primarily cell cultured. The area between 2.5 and 4.5 mm from the anterior tip of the rat brain was cut into coronal slices at the size of 400 μm with a microslicer.^28^ The slices were pre-incubated in solution that is well-saturated with 95% O_2_ – 5% CO_2_ gas at 25°C–27°C for 40 min.^28^ Then, the slices were incubated in 0.01–0.015% pronase at 31°C for 25 to 30 min and thereafter in 0.01% thermolysin under the same conditions.^28^ After that, the slices were kept in an enzyme-free incubation solution.^28^ The dorsal half of the neostriatum was micro-punched out and dissociated with fire-polished micro-Pasteur pipettes mechanically in a plastic culture dish filled with normal external solution.^28^ The dissociated neurons adhered to the bottom of the dish within 30 min for experiments of Na+, K + -ATPase current measurements at different step voltages.^28^

As listed in the literatures,^29^^,^^30^ single guinea pig ventricular myocytes were isolated from guinea pig ventricles. Adult guinea pigs at weights of 300–500 g were anesthetized with pentobarbital (∼50 mg/kg, i.p.) and then ventilated via a tracheotomy.^30^ The aortic arch was exposed and cannulated, the heart excised, and retrograde coronary perfusion begun, initially with normal Tyrode’s solution containing 3 U/mL heparin, then for 3 min with nominally Ca2+ free (∼1 μM free Ca2+ concentration) Tyrode’s, and then for ∼15 min with low Ca2+ concentration (∼5 μM) Tyrode’s containing 0.4–0.8 mg/mL collagenase.^30^ The collagenase was then washed out of the heart with high K+ concentration (∼160 mM), nominally Ca2+ free, solution containing 0.5 mM EGTA, 10 mM KH_2_PO_4_, 25 mM KCl, 20 mM taurine, 10 mM oxalic acid, 80 mM L-glutamic acid, 5 mM pyruvic acid, 10 mM dextrose, and 10 mM HEPES, all adjusted to pH 7.4 with KOH.^30^ All the above solutions were oxygenated and warmed to ∼36°C.^30^ The resulting partially digested heart was cut open and stored at 4°C in the high K+ concentration, Ca2+ free solution.^30^ Cells were transferred to the recording chamber, for experiments of Na+, K + -ATPase current measurements at different step voltages.^29^^,^^30^

### Method details

#### Cell model of ion flow by the stimulation

A cell with 6.6 μm diameter as shown in Figure 1 is stimulated by terahertz unipolar picosecond pulse train stimulation. The stimulation duration is 1.1 ns and 1.2 ns. The terahertz repetition frequency of the unipolar picosecond pulse train is respectively 0.1 THz, 0.21 THz, 0.3 THz, 0.5 THz, 0.51 THz, 0.7 THz, 0.9 THz and 1.2 THz. The duty cycle of the pulse train is 0.5 so the pulse width of each unipolar pulse in the train is 5 ps, 2.38 ps, 1.67 ps, 1 ps, 0.98 ps, 0.71 ps, 0.55 ps and 0.41 ps, respectively. Based on the frequency spectrum profile of the unipolar picosecond pulse train with terahertz repetition frequency,^19^ the electromagnetic frequency of those pulse train stimulation in terahertz range is mainly concentrated on the repetition frequency.

During the stimulation with those terahertz frequencies, the shortest wavelength of the terahertz electromagnetic wave in the cellular aqueous environment is far larger than the size of the cell (diameter of 6.6 μm) and also larger than the cell system in the simulation (diameter of 19.8 μm). It belongs to quasi-magnetostatic problem. And then according to the electromagnetic interaction theory between terahertz fields and physiological ions at the cellular level,^5^^,^^14^ the effects of magnetic fields can be ignored in the study of the life ion flow.

The capacitive current of the cell membrane is taken into account.^18^ The life ion flow in the intracellular and extracellular environments is driven by concentration gradients, and the ion flow across the cell membrane is depicted as follows.

##### Flow via the pores by the stimulation

The activation of cell membrane hydrophilic pores by the stimulation at the whole cellular level is numerically calculated with Neu and Krassowska electroporation model^18^^,^^19^^,^^31^ by,

(Equation 1) $$\left\{ \begin{array}{l} \frac{dN\left(t,\theta \right)}{dt}=\alpha e^{{\left(V_{m}\left(t,\theta \right)/V_{ep}\right)}^{2}}\left(1-\frac{N\left(t,\theta \right)}{N_{eq}\left(V_{m}\left(t,\theta \right)\right)}\right)\\ N_{eq}\left(V_{m}\left(t,\theta \right)\right)=N_{0}e^{q_{p}{\left(V_{m}\left(t,\theta \right)/V_{ep}\right)}^{2}} \end{array}\right.$$

where N(t, θ) is the density of hydrophilic pores in cell membrane at the polar angle θ at time t, V_ep_ is the characteristic voltage of hydrophilic pores, α is the creation rate of hydrophilic pores, N_eq_(V_m_(t, θ)) is the equilibrium density of hydrophilic pores at the membrane potential V_m_(t, θ), N_0_ is the equilibrium density of hydrophilic pores at zero membrane potential. q_p_ is the rate constant of the pore creation, q_p_ = (r_m_/r∗),^2^ r_m_ is the radius of the pore at the minimum energy at zero membrane potential, and r∗ is the minimum radius of the hydrophilic pores.

And the evolution of the radii of the hydrophilic pores is numerically evaluated by,^18^^,^^19^^,^^31^

(Equation 2) $$\frac{dr_{j}}{dt}=\frac{D_{p}}{k_{B}T}\left[\frac{{V_{m,j}}^{2}F_{\max}}{1+r_{h}/\left(r_{j}+r_{t}\right)}+4\beta {\left(\frac{r_{*}}{r_{j}}\right)}^{4}\frac{1}{r_{j}}-2\pi \gamma +2\pi \sigma_{eff}r_{j}\right],\left(j=1,2,...,K\right)$$

where r_j_ is the pore radius of the j^th^ hydrophilic pore, V_m,j_ is the membrane potential at the position of the j^th^ hydrophilic pore, D_p_ is the diffusion coefficient in the variation of the pore radius, k_B_ is the Boltzmann constant, T is the absolute temperature, F_max_ is the maximum electric force at V_m,j_ = 1, r_t_ and r_h_ are constants in the variation of pore radius, β is the steric repulsion energy, γ is the edge energy, σ_eff_ is the effective tension density of the membrane,^18^^,^^19^^,^^31^

(Equation 3) $$\sigma_{eff}\left(A_{p}\right)=2\sigma^{\prime }-\frac{2\sigma^{\prime }-\sigma_{0}}{{\left(1-A_{p}/A_{cell}\right)}^{2}}$$

where σ′ is the tension density at the interface between hydrocarbon chain of phospholipid molecules and water molecules, σ_0_ is the tension density of the phospholipid bilayer without hydrophilic pores in cell membrane, A_p_ is the total area of all the hydrophilic pores in the whole cell membrane, A_cell_ is the area of the whole cell membrane.

The membrane potential at the whole cellular level is numerically estimated based on the principle of current continuity at the whole cell membrane by,

(Equation 4) $$C_{m}\frac{\partial \left(\Phi_{i}-\Phi_{o}\right)}{\partial t}+g_{1}\left(\Phi_{i}-\Phi_{o}-V_{rest}\right)+I_{p}=0$$

and the Laplace equation of electrical potential by,

(Equation 5) $$\nabla^{2}\Phi =0$$

where C_m_ is the membrane capacitance, Φ_i_ and Φ_o_ are respectively intracellular and extracellular electrical potentials which are the functions of space and time, V_rest_ is the resting membrane potential, g_1_ is the membrane conductance of total physiological ion channels, I_p_ is the current of life ions via hydrophilic pores.

At the time and after the activation of the hydrophilic pores, each type of the life ion flow via the pores is numerically computed with the generalized modified Poisson-Nernst-Planck model^19^^,^^32^ by,

(Equation 6) $$J_{j}=-\frac{D_{j}c_{j}Fz_{j}}{R_{u}T}\nabla \Phi -D_{j}\nabla c_{j}-\frac{D_{j}c_{j}\sum_{j=1}^{N}N_{A}a_{j}^{3}\nabla c_{j}}{1-\sum_{j=1}^{N}N_{A}a_{j}^{3}c_{j}},\left(j=Na^{+},K^{+},Ca^{2+},Cl^{-}\right)$$

where F is Faraday constant, R_u_ is the gas constant, D_j_ is diffusion coefficient of the j type of ions, Z_j_ is the valence of the j type of ions, c_j_ is the ion concentration of the j type of ions, a_j_ is the effective ion radius of the j type of ions, ∇Φ is the gradient of electrical potential, N_A_ is Avogadro constant, N in the summation notation is the number of ion types, N = 4. The parameter values for the life ion flow via hydrophilic pores are listed in Table S1.

##### Flow via Na+, K + -ATPase by the stimulation

The Na+ and K+ flow via cell membrane Na+, K + -ATPase in the whole cell is numerically calculated with Na+, K + -ATPase model.^33^ The current of Na+, K + -ATPase in the whole cell is represented by,^33^

(Equation 7) $$I_{NaK-ATPase}=F_{c}\cdot v_{cyc}$$

where,

(Equation 8) $$v_{cyc}=\left(R_{1}^{+}R_{2}^{+}R_{3}^{+}R_{4}^{+}-R_{1}^{-}R_{2}^{-}R_{3}^{-}R_{4}^{-}\right)/\left(\begin{array}{l} R_{1}^{-}R_{2}^{-}R_{3}^{-}+R_{1}^{+}R_{2}^{-}R_{3}^{-}+R_{1}^{+}R_{2}^{+}R_{3}^{-}+R_{1}^{+}R_{2}^{+}R_{3}^{+}\\ +R_{2}^{-}R_{3}^{-}R_{4}^{-}+R_{2}^{+}R_{3}^{-}R_{4}^{-}+R_{2}^{+}R_{3}^{+}R_{4}^{-}+R_{2}^{+}R_{3}^{+}R_{4}^{+}\\ +R_{1}^{-}R_{3}^{-}R_{4}^{-}+R_{1}^{-}R_{3}^{+}R_{4}^{-}+R_{1}^{-}R_{3}^{+}R_{4}^{+}+R_{1}^{+}R_{3}^{+}R_{4}^{+}\\ +R_{1}^{-}R_{2}^{-}R_{4}^{-}+R_{1}^{-}R_{2}^{-}R_{4}^{+}+R_{1}^{+}R_{2}^{-}R_{4}^{+}+R_{1}^{+}R_{2}^{+}R_{4}^{+} \end{array}\right)$$

and,

(Equation 9) $$R_{1}^{+}=\frac{k_{1}^{+}{\left[\left(c_{Na\_i}/k_{d,Nai}^{0}\right)\exp\left(-\Delta_{Nai}FV_{m}/R_{u}/T\right)\right]}^{3}}{{\left[1+\left(c_{Na\_i}/k_{d,Nai}^{0}\right)\exp\left(-\Delta_{Nai}FV_{m}/R_{u}/T\right)\right]}^{3}+{\left[1+\left(c_{K\_i}/k_{d,Ki}^{0}\right)\exp\left(-\Delta_{Ki}FV_{m}/R_{u}/T\right)\right]}^{2}-1}$$

(Equation 10) $$R_{2}^{+}=k_{2}^{+}$$

(Equation 11) $$R_{3}^{+}=\frac{k_{3}^{+}{\left[\left(c_{K\_o}/k_{d,Ko}^{0}\right)\exp\left(-\Delta_{Ko}FV_{m}/R_{u}/T\right)\right]}^{2}}{{\left[1+\left(c_{Na\_o}/k_{d,Nao}^{0}\right)\exp\left(-\Delta_{Nao}FV_{m}/R_{u}/T\right)\right]}^{3}+{\left[1+\left(c_{K\_o}/k_{d,Ko}^{0}\right)\exp\left(-\Delta_{Ko}FV_{m}/R_{u}/T\right)\right]}^{2}-1}$$

(Equation 12) $$R_{4}^{+}=\frac{k_{4}^{+}c_{MgATP}/k_{d,MgATP}}{1+c_{MgATP}/k_{d,MgATP}}$$

(Equation 13) $$R_{1}^{-}=k_{1}^{-}c_{MgADP}$$

(Equation 14) $$R_{2}^{-}=\frac{k_{2}^{-}{\left[\left(c_{Na\_o}/k_{d,Nao}^{0}\right)\exp\left(-\Delta_{Nao}FV_{m}/R_{u}/T\right)\right]}^{3}}{{\left[1+\left(c_{Na\_o}/k_{d,Nao}^{0}\right)\exp\left(-\Delta_{Nao}FV_{m}/R_{u}/T\right)\right]}^{3}+{\left[1+\left(c_{K\_o}/k_{d,Ko}^{0}\right)\exp\left(-\Delta_{Ko}FV_{m}/R_{u}/T\right)\right]}^{2}-1}$$

(Equation 15) $$R_{3}^{-}=\frac{k_{3}^{-}c_{P}\cdot 10^{\left(3-pH\right)}}{1+c_{MgATP}/k_{d,MgATP}}$$

(Equation 16) $$R_{4}^{-}=\frac{k_{4}^{-}{\left[\left(c_{K\_i}/k_{d,Ki}^{0}\right)\exp\left(-\Delta_{Ki}FV_{m}/R_{u}/T\right)\right]}^{2}}{{\left[1+\left(c_{Na\_i}/k_{d,Nai}^{0}\right)\exp\left(-\Delta_{Nai}FV_{m}/R_{u}/T\right)\right]}^{3}+{\left[1+\left(c_{K\_i}/k_{d,Ki}^{0}\right)\exp\left(-\Delta_{Ki}FV_{m}/R_{u}/T\right)\right]}^{2}-1}$$

where F_c_ is a factor that is correlated with membrane capacitance and the number density of Na+, K + -ATPase protein channels in the cell membrane. And v_cyc_ is steady-state cycle rate of Na+, K + -ATPase. The factor F_c_ and the parameters in v_cyc_ are determined as the best fit values based on the whole-cell current-voltage experimental data of Na+, K + -ATPase. Those parameters in the v_cyc_ include the rate constants k_1_^+^, k_1_^-^, k_2_^+^, k_2_^-^, k_3_^+^, k_3_^-^, k_4_^+^, k_4_^-^, the dissociation constants for binding of Na+, K+ and ATP, k^0^_d,Nao_, k^0^_d,Nai_, k^0^_d,Ko_, k^0^_d,Ki_, k_d,MgATP_, and the coefficients that determine the voltage dependencies for the Na+ and K+ concentrations in the inside and outside of the cell Δ_Nai_, Δ_Ki_, Δ_Nao_, Δ_Ko_. The experimental data are measured in the cases of two different cell types, rat neostriatal neuron^28^ and guinea pig ventricular myocyte,^29^ so as to study the work of Na+, K + -ATPase and the energy consumption in these two cell types.

Suppose the positive direction is that a positive charge transports from the cell into the extracellular environment. Because Na+, K + -ATPase transports three Na+ ions from the cell into the extracellular environment in exchange for two K+ ions into the cell,^16^ the relationships between the life ion flow and the current of Na+, K + -ATPase are expressed by,

(Equation 17) $$J_{Na}=3I_{NaK-ATPase}/q_{ele}/N_{A}$$

(Equation 18) $$J_{K}=-2I_{NaK-ATPase}/q_{ele}/N_{A}$$

where J_Na_ and J_K_ are Na+ and K+ ion flow via Na+, K + -ATPase respectively. q_ele_ is the elementary charge (1.602 × 10^−19^ C). Then, the life ion flow at the polar angle θ in the spherical coordinate system (see in Figure 1) can be expressed by

(Equation 19) $$J_{Na\_NaK-ATPase}\left(\theta \right)=3I_{NaK-ATPase}/q_{ele}/N_{A}/A_{cell}\cdot A_{\theta }$$

(Equation 20) $$J_{K\_NaK-ATPase}\left(\theta \right)=-2I_{NaK-ATPase}/q_{ele}/N_{A}/A_{cell}\cdot A_{\theta }$$

where A_cell_ is the area of the whole cell membrane and A_θ_ is the element area at θ in the cell membrane. The parameter values for the life ion flow via Na+, K + -ATPase in the case of rat neostriatal neuron and guinea pig ventricular myocyte are respectively listed in Tables S2 and S3. And the current-voltage (I-V) response characteristics of rat neostriatal neuron and guinea pig ventricular myocyte at the initial ion concentrations listed in the Table S1 are illustrated in Figures 2A and 2B, respectively.

##### Power dissipation by the stimulation

While transporting Na+ and K+ ions, the Na+, K + -ATPase consumes ATP energy from cell metabolism. The hydrolysis energy of one ATP molecule is consumed to exchange three intracellular Na+ for two extracellular K+. Therefore, the consumption rate of ATP molecules is equal to one-third of the transport rate of Na+. Then, the Na+, K + -ATPase power dissipation of cell metabolic energy is the consumption rate of ATP molecules multiplied by the hydrolysis energy of one ATP molecule, which can be expressed by,

(Equation 21) $$P_{NaK-ATPase}=Q_{ATP}\cdot I_{NaK-ATPase}/q_{ele}/N_{A}$$

where Q_ATP_ is the hydrolysis energy of one ATP molecule (=10⋅k_B_T).

#### Numerical calculation methods

The simultaneous equations of the Laplace equation of electrical potential Equation 5, the ordinary differential equations of the electroporation model Equations 1, 2, and 3, the equation of ion flow of generalized modified Poisson-Nernst-Planck model Equation 6, and the ordinary differential equation of membrane potential in terms of transmembrane ion current with the considerations of the capacitive current Equation 4 are numerically solved by finite difference in time domain. In the calculation of the electrical potentials, the electric charges of each type of ions in bulk solution are neglected since the net charges of all the ions in the intracellular and extracellular bulk solution are both approximately 0.^14^^,^^18^ The resting potential of the cell arisen from ions in the solution is represented by the initial conditions in the calculation of the electrical potentials.

The Na+ and K+ flow via Na+, K + -ATPase is calculated by (Equation 7), (Equation 8), (Equation 9), (Equation 10), (Equation 11), (Equation 12), (Equation 13), (Equation 14), (Equation 15), (Equation 16), (Equation 17), (Equation 18), (Equation 19), (Equation 20), and the power dissipation is estimated by Equation 21. The ion flow and ion concentration are alternately calculated to get the power dissipation as well as the transmembrane life ion flow and the ion concentration at any time.

### Quantification and statistical analysis

The numerical calculation program in this study is written in C language in Visual Studio 2015 Version and the results are plotted by writing codes in MATLAB software. The programs in C language have been deposited at Zenodo (https://zenodo.org/deposit/8252966), and the programs in MATLAB have been deposited at Zenodo (https://zenodo.org/deposit/8252966).

## Data Availability

• Data of the power dissipation, intracellular life ion concentrations, life ion fluxes via cell membrane hydrophilic pores and Na+, K+-ATPase as well as the membrane potential and membrane conductance with respect to time have been deposited at Zenodo (https://zenodo.org/record/8252966) with DOI https://doi.org/10.5281/zenodo.8252966 and are publicly available as of the date of publication. • All original code for the calculations in the numerical calculation methods in STAR Methods with c language and the plotting of the results with Matlab has been deposited at Zenodo (https://zenodo.org/record/8252966) with DOI https://doi.org/10.5281/zenodo.8252966 and is publicly available as of the date of publication. • Any additional information required to reanalyze the data reported in this paper is available from the lead contact upon request.

## Supplemental information

Document S1. Tables S1–S3 Table S1. Parameter values for the life ion flow via cell membrane hydrophilic pores by the stimulation. Table S2. Parameter values for the life ion flow via Na+, K+-ATPase in the case of rat neostriatal neuron. Table S3. Parameter values for the life ion flow via Na+, K+-ATPase in the case of guinea pig ventricular myocyte.
