# Supplementary material for: Investigations on Na+, K+-ATPase energy consumption in ion flow of hydrophilic pores by THz unipolar stimulation
Source: iScience. 2023 Sep 7;26(10):107849. doi: 10.1016/j.isci.2023.107849 (PMC10520936; doi:10.1016/j.isci.2023.107849)
Supplement: Document S1. Tables S1–S3 — Table S1. Parameter values for the life ion flow via cell membrane hydrophilic pores by the stimulation. Table S2. Parameter values for the life ion flow via Na+, K+-ATPase in the case of rat neostriatal neuron. Table S3. Parameter values for the life ion flow via Na+, K+-ATPase in the case of guinea pig ventricular myocyte. [file mmc1.pdf]

iScience, Volume *26*

Supplemental information

Investigations on Na+, K+-ATPase energy consumption in ion flow of hydrophilic pores by THz unipolar stimulation

Wenfei Bo, Rong Che, Qiang Liu, Xiaobo Zhang, Yintao Hou, and Yubin Gong

Table 1

parameter	value	parameter	value
V_{ep}	0.258 V	N_A	6.022×10^{-23} /mol
α	1×10^{9} m^2/s	D_{Na}	1.33×10^{-9} m^2/s
N_0	1.5×10^{9} $/m^2$	D_K	1.96×10^{-9} m^2/s
r_m	0.8×10^{-9} m	D_{Ca}	1.4×10^{-9} m^2/s
r_*	0.51×10^{-9} m	D_{Cl}	2.07×10^{-9} m^2/s
D_p	5×10^{-14} m^2/s	Z_{Na}	1
k_B	1.3806×10^{-23} J/K	Z_K	1
T	309.15 K	Z_{Ca}	2
F_{max}	0.70×10^{-9} N/V^2	Z_{Cl}	-1
r_t	0.31×10^{-9} m	a_{Na}	0.358×10^{-9} m
r_h	0.97×10^{-9} m	a_K	0.331×10^{-9} m
β	1.4×10^{-19} J	a_{Ca}	0.412×10^{-9} m
γ	1.8×10^{-11} J/m	a_{Cl}	0.332×10^{-9} m
σ'	2×10^{-2} J/m^2	c_{Na_i} (initial)	12 mol/m^3
σ_0	1×10^{-3} J/m^2	c_{Na_o} (initial)	145 mol/m^3
A_{cell}	1.3685×10^{-10} m^2	c_{K_i} (initial)	139 mol/m^3
C_m	1×10^{-2} F/m^2	c_{K_o} (initial)	4 mol/m^3
V_{rest}	-80 mV	c_{Ca_i} (initial)	1×10^{-4} mol/m^3
g_l	2.0 S/m^2	c_{Ca_o} (initial)	1.8 mol/m^3
F	9.6485×10^{4} C/mol	c_{Cl_i} (initial)	4 mol/m^3
R_u	8.314 J/(mol·K)	c_{Cl_o} (initial)	116 mol/m^3

Table 2

parameter	value	parameter	value
F_c	4.244×10^{-3}	$k^0_{d,Ko}$	0.6084
k_1^+	2.088	$k^0_{d,Ki}$	0.6787
k_1^-	0.01479	$k_{d,MgATP}$	0.008932
k_2^+	0.7648	Δ_{Nai}	-2.088
k_2^-	8.54×10^{-5}	Δ_{Ki}	-2.843
k_3^+	0.2698	Δ_{Nao}	0.2316
k_3^-	1.472	Δ_{Ko}	0.1364
k_4^+	3.129×10^{-8}	c_{MgATP}	6.9 mol/m^3
k_4^-	0.0971	c_P	3.6 mol/m^3
$k^0_{d,Nao}$	0.4982	c_{MgADP}	1.2 mol/m^3
$k^0_{d,Nai}$	5.034	pH	7.4

Table 3

parameter	value	parameter	value
F_c	5.850×10^{-9}	$k^0_{d,Ko}$	0.8
k_1^+	0.72	$k^0_{d,Ki}$	18.8
k_1^-	0.08	$k_{d,MgATP}$	0.6
k_2^+	0.08	Δ_{Nai}	-0.14
k_2^-	0.008	Δ_{Ki}	-0.14
k_3^+	4	Δ_{Nao}	0.44
k_3^-	8000	Δ_{Ko}	0.23
k_4^+	0.3	c_{MgATP}	6.9 mol/m^3
k_4^-	0.2	c_P	3.6 mol/m^3
$k^0_{d,Nao}$	26.8	c_{MgADP}	1.2 mol/m^3
$k^0_{d,Nai}$	5	pH	7.4
